# Cancer Stem Cells: From Historical Roots to a New Perspective

**DOI:** 10.1155/2019/5189232

**Published:** 2019-06-11

**Authors:** Jean-Pascal Capp

**Affiliations:** INSA/University of Toulouse, Laboratoire d'Ingénierie des Systèmes Biologiques et des Procédés, UMR CNRS 5504, UMR INRA 792, Toulouse, France

## Abstract

The relationships between cancer and stemness have a long history that is traced here. From the mid-19th century when the first theory on the embryonic origin of cancer was formulated to works on embryonal carcinoma cells in the mid-20th century, many steps have been crossed leading to the current cancer stem cell theory postulating that tumor growth is supported by a small fraction of the tumoral cells that have stem-like properties. However, in the last fifteen years, many works regularly encourage us to revise the concept of cancer stem cell. This article mentions key results that lead to a new perspective where cancer stem cells are primarily seen as cells exhibiting increased epigenetic plasticity and increased gene expression variability. This perspective suggests new therapeutical interventions consisting in stabilizing gene expression to control cancer cell proliferation and prevent stochastic gene expression variations that could lead to therapeutic resistance.

## 1. Historical Roots

### 1.1. From the 19th Century to the Middle of the 20th Century

It is possible to date the first mention of the role of undifferentiated cells in cancer in the 1870s, with the debate between Rudolf Virchow (1821-1902) and his student Julius Cohnheim (1839-1884). Cohnheim extensively formulated in 1877 his theory of the embryonic origin of cancer, which postulates that the origin of tumor development must be attributed to the existence in the body of “embryonic rests” that have remained unused during development [1]. This idea was not revolutionary in itself because, as early as 1838, Johannes Müller (1801-1858) had described tumors as the abnormal continuation of embryonic cell development on the basis of morphological similarities. Virchow himself had emphasized the correspondence between embryonic and tumor development, indicating that these processes are both derived from cell division and multiplication [1]. But Cohnheim went further than the morphological similarities by imagining a common origin of all tumors based on the presence of persistent embryonic cells in the body. According to him, if these cells receive the necessary blood supply, they begin to proliferate uncontrollably because of their embryonic nature. They subsequently form tumors that are considered as a consequence of “errors” during development [2]. Therefore tumors would be the result of the high proliferation propensity of these embryonic rests. These rests would also explain why various mature cell types can be observed [1]. Finally Cohnheim additionally mentioned that these embryonic cells can also be the origin of the normal cell proliferation observed in physiological cases during puberty or pregnancy [1].

Cohnheim's theory was much discussed at the end of the century and considered as a real alternative to the parasitic or chemical theories of cancer. Experiments have tried to demonstrate its validity, with limited success because the reimplanted embryonic cells differentiated very often and behaved normally. However, Max Askanazy (1865-1940) was then able to obtain in rats tumors that resembled teratomas (tumors that contain differentiated elements of all three embryonic germ layers and that occur most commonly as benign ovarian tumors, dermoid cysts, and, rarely, as tumors of newborns) [2] the tumor type on which Cohnheim based his generalization. Thus, teratomas became the preferred model for understanding the formation of all tumors (although Virchow considered it an exception [1]), but also for understanding normal cell proliferation phenomena in adults. In 1907, Askanazy used the term stem cells (Stammzellen) to designate these cells as embryonic remnants that should be discarded in the early stages of development and whose maturation was delayed or stopped [2]. It is interesting to note that Hugo Ribbert (1855-1920), professor of pathology in Bonn, formulated a modified version of Cohnheim's theory considering that sequestration of undifferentiated cells could take place not only during development, but also during the life of the individual because such cells could be generated if a lack of “tissue tension” appears. It would trigger the proliferation of these cells in their new environment [2]. On the contrary, if cells are maintained in their normal physiological context within a network of tissue interactions, their proliferation capacity would be counteracted by this tension.

Finally, Theodor Boveri (1862-1915) was also interested in cancer by observing that the abnormal distribution of chromosomes during cell division causes the loss of proliferation-inhibiting phenomena and leads to abnormal behavior of the daughter cells that he has assimilated to the behavior of cancer cells. Based on these observations, he formulated the first chromosomal theory of cancer (see [3] for the genesis of the first theories of cancer). From this perspective, he considered that in most cases the immature characteristics of cancer cells are side effects of this abnormal distribution of chromosomes and that embryonic rests can be incriminated only in rare cases. These ideas had a great influence because tumor cells have been considered during the following decades as well-differentiated cells that have become dedifferentiated. This was the opposite of Cohnheim's conception. It was only in the 1960s and 1970s that new works on teratocarcinomas (malignant tumors that are more aggressive than teratomas and formed of undifferentiated cells and differentiated cells of all three embryonic germ layers) renewed in a different modern context the question of the role of stem cells in the genesis of cancers.

### 1.2. Study of Teratomas, Teratocarcinomas, and Embryonic Carcinomas

As mentioned above, Julius Cohnheim based his theory of the embryonic rest on the study of teratomas. It is logically also that, based on teratomas, on the more aggressive tumor type teratocarcinomas, and on the very aggressive cancer embryonal carcinoma consisting only in poorly differentiated embryonal carcinoma (EC) cells, efforts were made to characterize the potential role of undifferentiated cells in tumorigenesis [4, 5]. As early as 1953, a study of one thousand cases of testicular tumors had concluded that, in most cases, undifferentiated germinal cells are the origin of tumor development because cells in these tumors possess potentialities similar to those of the germinal cells [6]. However, their exact nature remained to be identified. In the same year, Leroy Stevens (1920-2015) began working in Clarence C. Little's (1888-1971) laboratory on the effect of mutagenic and carcinogenic agents in various mouse lines. By systematically examining many mice, he found that the line called “129” was particularly sensitive to testicular teratomas, whereas this type of tumor had only been described in very rare cases in mice. In 1954, Stevens and Little reported thirty cases out of 3557 male “129” mice examined, about 1% [7]. In addition, these tumors were found to be transplantable in series because the injection of tumor material under the skin or into the abdominal cavity of new mice has maintained tumor growth over sixteen generations. For the authors, these tumors were thus composed of rapidly dividing undifferentiated cells of embryonic type [7].

Other subsequent studies on the “129” line showed that these spontaneous tumors originate in the seminiferous tubes [8], that the transplantation of teratomas into the mouse's abdomen forms “embryonic-like bodies” (or embryoid bodies) [8, 9], and that, conversely, some embryonic tissues, or even fertilized eggs, are capable of generating teratomas and teratocarcinomas when they are implanted in the testis of mice [10–12]. The second point has shown that these tumors have more in common with the embryo development than Stevens originally thought because the embryoid bodies have an organization in endoderm and ectoderm similar to the one of the embryo [13]. But the last point is essential because, contrary to the initial hypothesis that these tumors develop from poorly differentiated germ cells, it showed that highly undifferentiated cells of the early embryo, and not just primordial germ cells, are capable of generating teratomas. Stevens referred to this type of cell as a “pluripotent embryonic stem cell” (the origin of the term embryonic stem (ES) cell [1]), a term that was long interchangeable with that of EC cells [1, 2].

G. Barry Pierce (1925-2015) at the University of Michigan was more interested in embryonal teratocarcinomas and carcinomas in the early 1960s and showed in 1964 that EC cells (derived from these two types of cancer) produce various differentiated tissues and embryonal carcinomas when transplanted in mice [14]. He interpreted this observation as supporting the theory of cancer stem cells (CSC) and showed at the same time that EC cells were multipotent [14]. These cells can be maintained in an undifferentiated state* ex vivo* or by transplantation in the animal, while maintaining a set of chromosomes that appears normal [4, 13]. But they can also be intentionally differentiated* ex vivo*, and their resemblance to the normal embryonic cells contained in the internal mass of late blastocysts led to studies showing that EC cells, when injected into early embryos (at the blastocyst stage), are able to contribute to the normal development of “chimeric” animals [15–17]. Finally, it is remarkable that Pierce, from the end of the 1960s, relied on these results to formulate a theory of cancer where tumors would be formed by “induction” of nongenetic origin or by disruption of the “developmental field”, rather than by mutation in a single cell which would then multiply anarchically [18]. This would make it a reversible phenomenon. This conception needs more than ever to be reconsidered as a real alternative to the theory of somatic mutations [3] (see below). Thus, the 1960s brought the modern era of stem cell research through a curious mix of studies on physiological and pathological phenomena that intertwined and nourished each other.

### 1.3. From Murine EC Cells to Human ES Cells

In the late 1960s and the 1970s, several groups were able to establish the first murine EC cell lines and pave the way for their molecular characterization [4]. Then it was in the early 1980s that human lines of such cells were obtained. From the beginning, the isolation and characterization of EC cell lineages depended on specific molecular markers that must be identifiable in living cells [4]. The first marker used was the expression of the enzyme called alkaline phosphatase, which is strongly expressed in EC cells [5]. But it was the development of monoclonal antibodies in the 1970s that led to the development of molecular detection of these cells. For example, the Forssman and SSEA1 (stage-specific embryonic antigen 1) antigens are specific for murine EC cells, with the latter not reacting with those of human origin [5]. On the other hand, two other antigens, SSEA3 and SSEA4, have been found to be specific for human EC cells [5]. In the absence of direct studies on human embryonic cells because of the lack of an established lineage, it was supposed that the results on human EC cells could be extrapolated to what happens in the early embryo. But the fact that human and murine EC cells do not have the same characteristics made this extrapolation uncertain [4].

EC cells can be obtained from mouse embryos, but indirectly after transplantation of embryo tissues into an ectopic location, then development of a teratocarcinoma, and finally isolation of EC cells from this teratocarcinoma. The next obvious step was to obtain similar cells directly derived from embryos, i.e., true murine ES cells [5]. The first mouse ES cell lines were independently isolated by two research groups from* ex vivo*-grown blastocysts [19, 20]. This was a decisive step as they were isolated without the production of teratocarcinomas. These mammalian ES cells were isolated from the internal mass before separation of the epiblast and the primitive endoderm. But the precise correspondence between the ES cells obtained and grown* in vitro*, and their equivalent in the embryo have long been controversial. It now appears that ES cells are more similar to epiblast cells than to cells in the early internal mass and that pluripotency is acquired during epiblastic type formation [21]. Nevertheless, studies have confirmed the similarity between ES and EC cells, in terms of molecular markers and ability to differentiate* ex vivo* and* in vivo* [5]. Moreover, in addition to their embryonic features, ES cells are tumorigenic and form teratocarcinomas when injected into an adult organism. Since these teratocarcinomas are indistinguishable from those generated by EC cells, this was also considered as an important criterion for the embryonic nature of ES cells [13].

Works on murine ES cells were initially used rather to create protocols of production of transgenic mice, thanks to the introduction of precise mutations into their genome* ex vivo* in order to study their effects in animals, than to study the biology of these cells and* ex vivo* differentiation processes [4, 13]. Gene inactivation in these cells by the introduction of a nonfunctional copy has been a key issue in the 1980s [13] and a major step was crossed when it was possible to effectively select ES cells in which these rare events occurred [22, 23]. Other mammalian ES cell lines were obtained during the 1980s, but primate ES cells were cultured only in 1995 [24].

Finally, the production of the first lines of human ES cells arrived in 1998 thanks to two groups that independently isolated them from aborted embryos [25] or embryos fertilized* in vitro* [26]. These works may seem late when considering the availability of markers necessary for the identification of human ES and EC cells, and of ES cell isolation techniques that have been established for a long time for mice and are comparable for human cells. This delay can be explained by difficulties in obtaining human embryos allowing this isolation and especially by the reluctance of many researchers on works that cause problems of legal nature, and political and moral dilemmas.

### 1.4. Reemergence of the Cancer Stem Cell Concept: Molecular and Cellular Aspects

Another phenomenon has greatly contributed to the need of molecular characterization of stem cells: the reemergence of the concept of CSC and its role in carcinogenesis [27]. Differentiation problems in tumors were somewhat neglected in the 1980s in favor of the effects of oncogenes on proliferation and genome stability. This was still the case in the early 1990s, especially because many researchers have considered teratocarcinomas, which served as a model for studying these problems of differentiation, as a particular case of no great interest for the study of other cancers. It is the study of certain forms of leukemia, in particular acute myelogenous leukemia (AML), that put the concept of CSC at the forefront [27].

As such, the studies of John E. Dick's lab and colleagues published in 1994 and 1997 were crucial as they demonstrated that only few rare cells of mouse AML are capable of initiating this leukemia in other mice. Moreover, they showed by series of successive transplants that these cells have a very high capacity for self-renewal, which is an essential characteristic of stem cells [28, 29]. The tumors that appear after transplantation are composed of a mixture of tumorigenic cells and nontumorigenic cells similar to the one of the initial tumor [29]. This shows that the same process is reproduced at each cancer development: CSC produce both cells identical to those that were transplanted (by self-renewal) and more differentiated cells that lose their tumorigenic potential by a process of differentiation. These CSC have now been identified in many types of cancers, including solid tissues [30]. Indeed, the presence of CSC in solid tumors was demonstrated in the 2000s first in breast cancers where as few as hundred CSC were able to form tumors in mice, whereas tens of thousands of cells with alternate phenotypes failed to form tumors [31]. Then works on brain and colon cancers further identified such subpopulations of rare cells that produce tumors* in vivo* [32, 33].

In many ways, the CSC identified in AML were close to normal blood stem cells. This enhanced the interest in trying to characterize their molecular features. But this interest was also based on the desire to find what distinguishes CSC from normal stem cells and what would allow targeting them for therapy without affecting normal cells. Conversely, this has also contributed to better characterizing of normal stem cells [34].

At the same time, the CSC hypothesis was refined. It now postulates that tumors are hierarchically organized, just like normal tissues. They would be maintained by a fraction of cells responsible for tumor formation and growth. These cells (also known as “cancer-initiating cells” or “cancer stem-like cells”) possess key characteristics of normal stem cells, namely, self-renewal, unlimited proliferation but infrequent divisions, and an ability to produce offspring capable of differentiation. However, these CSC would generate cells that only partially differentiate and whose differentiation appears to be stopped at intermediate stages. Here again, this blockage has contributed to further study of the differentiation of normal stem cells. As with normal stem cells, the niche concept has also been applied to CSC. This would be a specific environment necessary for the survival and maintenance of their properties [35]. Finally, many factors have led researchers interested in stem cells to characterize the signaling pathways that maintain stemness, the surface markers that allow their isolation, or the networks of transcription factors that form and generate their phenotypic identity. But this view of the stem cell based on a specific and fixed identity soon proved to be insufficient, or even largely false. The 2010s have indeed changed our conception of stemness. These changes have a fundamental impact on the conception of differentiation processes, but must also be considered in oncogenesis.

## 2. A new Perspective on Cancer Stem Cells

### 2.1. Stem Cells: State rather than Entity

ES cells possess an unusual nuclear structure where DNA appears to be packaged in a more open chromatin structure than in differentiated cells. In the mid-2000s, this was associated with the fact that chromatin proteins are more rapidly exchanged at the DNA level, reflecting their weaker interactions [36]. In addition, ES cells show an enrichment in epigenetic marks associated with high gene expression activity and possess fewer of those that make chromatin too compacted to allow expression [37]. It remained to analyze the real impact of this particular chromatin structure on gene expression.

Tom Misteli's team published in 2008 an essential work that deeply contributed to the change of our vision of stemness [38]. It showed that mouse ES cells express their genes in a generalized and widespread manner and that differentiation is associated with large-scale repression of gene expression at the genomic level. Gene expression profiles clearly become more discontinuous in differentiated cells. The “hyperactivity” of ES cells in terms of gene expression is accompanied by the presence in unusually high levels of proteins involved in chromatin remodeling and gene transcription. Moreover almost all of the tissue-specific and differentiation genes that have been examined are sporadically expressed at a low level. Thus eleven out of twelve genes associated with terminal differentiation and therefore not expected in ES cells have been found to be expressed at 0.25 to 20 RNA copies per cell, suggesting a strong stochastic appearance of this expression [38].

Stochasticity in gene expression was previously demonstrated in the early 2000s in microbial populations thanks to the development of single cell analysis by flux cytometry and to the construction of synthetic gene networks that led to the discovery of large fluctuations in gene expression levels among individual cells in isogenic populations [39, 40]. The term noise in gene expression is now used to designate this variation in the expression level of a gene under constant environmental conditions [41]. It is also used to quantify the heterogeneity in expression from cell to cell by dividing the variance across the population by the mean [42].

It should be noted that this generalized transcriptional activity had already been observed in hematopoietic stem cells where the expression of differentiation genes was noticed before any differentiation, with a large portion of the genome expressed in the undifferentiated state [43]. However this new study on ES cells has had a strong impact and must be associated with the work from Sui Huang's laboratory published also in 2008. It focused on progenitor cells of the hematopoietic system [44]. Two major observations were made. On the one hand, these cells very heterogeneously express the stem cell-associated Sca-1 gene, and when a small homogeneous fraction of this population is isolated and cultured again, it spontaneously returns to the original heterogeneity after few days. On the other hand, the expression or absence of expression is related to a propensity to differentiate towards one pathway or the other in the hematopoietic system. This reflects an important bias related to this expression. Then many other articles have confirmed and reinforced these first results and brought interesting results. In particular, gene expression variability in ES cells* in vitro* depends on the culture conditions and therefore on the cellular environment [45].

Thus stem cells cannot be defined by a stable phenotype. They are in a state of permanent instability and are not defined by the stable expression of few specific genes. Some transcription factors considered to be specific to ES cells, such as the pluripotency-involved Nanog protein [46] or various other “markers” [47], are expressed with high heterogeneity. They seem to fluctuate between high and low levels of expression, even if a clustering into more or less activated subgroups is possible [48]. A detailed study of the behavior of the Nanog protein has led to the conclusion that its variations are an essential element of the pluripotent state [49]: cells randomly fluctuate between a stable state of high Nanog expression and another state with low Nanog expression which is highly unstable, giving rise to heterogeneous cell populations. Noise-driven fluctuations can lead individual cells to stochastic excursions into this transient state with low levels of Nanog where they are more likely to differentiate depending on the culture medium [49]. Thus pluripotency seems dependent on the stochastic expression fluctuations of a panel of genes, and the role of the gene regulatory network centered on Nanog could be to generate this heterogeneity [49]. Similarly, mouse ES cells that express less Nanog have a stronger tendency to differentiate, and cells with higher Nanog levels have a more stable gene expression pattern under various culture conditions and minimal expression of differentiation genes [50]. Nevertheless, other studies suggest that differences in the expression of Oct4, Nanog, or c-Myc proteins in mouse ES cells poorly affect pluripotency [51, 52], although, again, the propensity to differentiate depends on their level of expression [49, 52].

As mentioned below, there is a generalized and highly stochastic gene expression, including that for differentiation genes, in ES cells, and the observed heterogeneity is reconstituted from a homogeneous subpopulation that would have been isolated. But these heterogeneous subpopulations have varying inclinations for various differentiation pathways. These works are contradictory to what was postulated in the model that prevailed in the early 2000s when the finely regulated stem cell was supposed to express only few specific genes in a homogeneous manner, reacting also in a homogeneous way to differentiation “signals”. Very few researchers had a different conception. Dov Zipori, who based his analysis mainly on hematopoietic cells, had suggested in 2004 that it actually seems impossible to define a molecular signature of stem cells and that generalized and variable gene expression defines these cells [53]. Moreover, proteins associated with differentiated cell types are expressed with high heterogeneity* in vivo* in early mice embryos [54], and differentiation would thus have be considered as the suppression of this generalized and variable gene expression [55]. Also, this has to be correlated with the transition from a very open and dynamic chromatin to a more stable and closed state [56]. The expression level of the vast majority of genes indeed decreases during differentiation [55].

An intriguing hypothesis explaining the heterogeneity and the epigenetic instability of stem cells is related to energy metabolism. Indeed, a whole set of experiments indicates many connections between pluripotency and their metabolic activity [57, 58]. For example, stimulation of glycolysis by chemical agents [59] or cell culture under oxygen-poor conditions [60] allows dedifferentiation of differentiated cells into pluripotent cells. Inhibition of glycolysis or stimulation of oxidative phosphorylation has the opposite effect [59, 61]. This is consistent with the fact that ES cells differentiation is associated with a metabolic switch from a glycolytic type to an oxidative type [62].

The link between the highly dynamic chromatin status in pluripotent cells and metabolism comes from the observation that the latter produces molecules that are used for chromatin modification reactions. Actually metabolism emerges as a key player in epigenetics, with metabolites such as acetyl-coA, S-adenosylmethionine, or *α*-ketoglutarate among others that are cofactors used by chromatin modifiers [63, 64]. The cellular amount of these key metabolites, which reflects the metabolic state of the cell, clearly affects the chromatin state with direct consequences on the expressed portion of the genome. Thus, fluctuations in their concentration as a function of the metabolic activity would directly result in global changes in chromatin opening and thus in changes in the level of expression variability [65]. A metabolism favoring the production of molecules such as acetyl-coA used in reactions that open up chromatin (histone acetylation) would allow a more generalized and stochastic gene expression.

Finally, it was proposed in 2013 to be applied to pluripotency statistical methods used in thermodynamics [66]. Indeed, the permissive chromatin state in stem cells means that stemness cannot be well-defined at the single cell level. Functional pluripotency spontaneously emerges from dynamic variability intrinsically linked to the pluripotent state. This variability would be spatiotemporally regulated during* in vivo* development, but these restrictions would be largely absent* in vitro* and the intrinsic variability of the population would become apparent. From this perspective, the highly variable panel of gene expression in stem cells is expected to allow many developmental choices. On the contrary, the appearance of constraints during differentiation (see below) makes it possible to obtain less various and better defined expression panels so that the differentiated state is characterized by stable phenotypes. This process is linked to the progressive closing of the chromatin.

### 2.2. Cancer Stem Cells: A Controversial Theory

The current CSC theory postulates that a small proportion of tumor cells are able to support their growth because they provide dividing daughter cells whose differentiation is incomplete, which proliferate rapidly (unlike CSC) and which have a limited number of possible divisions and form the majority of the tumor. In fact, these daughter cells do not self-renew and are unable to form new tumors in healthy animals, whereas these two points are the functional definition criteria for CSC.

This concept of CSC allowed, on the one hand, better understanding and explaining the heterogeneity of cells within tumors. Indeed, poorly differentiated cells such as CSC seem better able to generate very heterogeneous cell populations by uncontrolled differentiation phenomena than differentiated cells that dedifferentiate. This phenomena is especially evident when looking at the modification of the balance between symmetric and asymmetric divisions of CSC during cancer progression. Early stage tumors are characterized by asymmetric division of CSC to form well-differentiated tumors composed of a mix of differentiated states, while late-stage cancer suppresses asymmetric division, which promotes symmetric self-renewal to form undifferentiated tumors [67, 68]. On the other hand, the concept of CSC allows considering otherwise the emergence of therapeutic resistance. CSC are more likely to escape chemo- or radiotherapeutic treatment and can transmit this resistance to their offspring. Recurrent cancers from these CSC are therefore more resistant to treatment than primary tumors. This phenomenon has already been observed in many cancers like breast cancers where, following chemotherapeutic treatment, secondary tumors showed a significant increase in the proportion of CSC since only the other cells were killed by chemotherapy [69]. CSC would therefore be responsible for the recurrence of the tumor, after a period of apparent remission [70]. The same phenomenon has also been demonstrated in glioblastomas, for instance, [71]. Finally, in addition to tumor recurrence, CSC are also suspected to be key drivers in metastatic dissemination [72].

Other studies have also reinforced the CSC theory. Those mentioned so far have revealed that only a subpopulation of cancer cells is able to form new tumors in immunodeficient animals, but none had shown the role of CSC in the development of solid endogenous tumors until 2012. Three studies on brain [71], intestinal [73], and skin [74] tumors used* in vivo* cell line tracing techniques to demonstrate the existence of CSC, concluding that it is necessary to target and eliminate CSC to eradicate cancer. But this is a real challenge because of the low proliferation of these cells (chemo- and radiotherapy most often affect cells that actively proliferate).

Another major issue in this field is the cell of origin in cancers: is cancer caused by a normal stem cell or a differentiated cell that is dedifferentiated? The hypothesis of normal stem cells was reinforced by a study describing the mathematical correlation between the frequency of appearance of cancers in some tissues and the frequency of cell division at the basis of the renewal of these tissues, and therefore the frequency of mutations that appear over the divisions in these cells [75]. However, both hypotheses seem possible [76, 77], even if mutations in known oncogenes have only a very limited impact on the expansion of stem cells that are in their niche [78]. Indeed, on the one hand, these common mutations confer only a very limited competitive advantage and the mutated cells are easily and stochastically replaced by “normal” equivalents from neighboring cells. On the other hand, mutations within osteoblastic cells which constitute, among other cells, the niche of hematopoietic stem cells, are sufficient to generate leukemia [79, 80]. It seems that it is the interactions of the stem cells with their niche, rather than their genetic content, that matters for cancerous development.

Beyond their origin, the question of their proportion in the tumor and their characteristics raises acute problems in the current framework. This was highlighted in 2008 by a study which, while it was thought that CSC constituted 0.0001% to 0.1% of the tumor mass, identified up to 25% of CSC when melanoma cells were transplanted into immunodeficient mice [81]. In addition, no specific molecular marker was identified to differentiate them from other tumor cells. This major difference in CSC frequency would be related to the environment in which cells try to proliferate because the same authors showed that a protocol identical to the one used in a previous study gave the same low frequency of tumorigenic cells (0.0001%) [82]. On the other hand, this proportion is increased by various modifications of the original protocol (lengthening of the period of observation, injection with an environment rich in components of the extracellular matrix to increase cell viability, or host more immunodeficient). The CSC frequency therefore depends on the method used [81] and cell-cell interactions seem to play a crucial role. It is clear that this greatly complicates the CSC theory [83] or even puts it in difficulty. Cells that are apparently nontumorigenic can become tumorigenic in the presence of the appropriate microenvironment. Thus, being too rigid in the way of defining CSC is not realistic [83].

In addition, at least some markers used to identify CSC are not stable. For instance, within human melanoma cells, only one subpopulation expresses the JARID1B enzyme [84] and, compared to melanoma cells that do not express it, this subpopulation does not proliferate much, which seems to make it fall into the category of CSC. But intriguingly, the expression of the JARID1B enzyme, which seems to be a CSC marker, is dynamic: its expression can frequently appear in cells that did not express it initially. The stem state could therefore be acquired by any cell at any time, rendering difficult any therapeutic intervention targeting CSC [85]. This plasticity of the CSC state has also been recently demonstrated in glioblastoma [86]. Conversely, glioblastoma CSC can express various differentiation markers and still contribute to tumor initiation and self-renewal [87]. This has led some researchers to propose a stochastic model of CSC where each cell of a tumor has the ability to act as CSC [27, 88]. Variations in this ability are due to intrinsic variations in the cells and to the environment in which these cells are located. (In the classical hierarchical model, when a cell leaves the stem state, the phenomenon is irreversible and none of its descendants can restore it.)

The latest versions of the CSC theory evoke a “tumor reprogramming” due to genetic alterations initiating cancer that would “reset” the epigenetic status and gene expression in initially healthy cells [89]. Thus, oncogenes would contribute less to the development of cancers by inducing cell proliferation than by causing “developmental reprogramming” of the epigenome of the affected cell [90]. This would allow these cells to aberrantly and pathologically differentiate. The parallelism with the cell reprogramming process which aims at producing induced pluripotent stem cells (iPS cells) is therefore striking. In addition, the initial genetic events that would have the main role of causing this reprogramming would become, like in iPS cell generation, useless for tumor progression when reprogramming is complete. This would explain why these alterations are no longer maintained or commonly observed [91]. However, the origin of early dedifferentiation in cancer may be considered otherwise by focusing on the tissue level [92–94] (see below). This would resolve many paradoxes concerning, for example, the widespread presence of so-called oncogenic alterations (that is to say, supposed to cause cancer) in healthy tissues, such as in the skin [95] or in the oesophagus [96].

Finally, metabolism has emerged as a key player in gene expression and epigenetics also in cancer cells [97]. More specifically, some reports showed that CSC preferentially use glycolysis while other works reported a propensity for mitochondrial oxidative phosphorylation suggesting a possible metabolic plasticity [98, 99]. In any case, it is entirely possible that the epigenetic plasticity that characterizes cancer cells and drives tumor adaptation [100] occurs mainly through metabolic modifications in CSC that directly impact epigenetics. Their specific metabolic state probably makes them very plastic in terms of gene expression with a more widespread and stochastic expression of the genome as in ES cells.

### 2.3. Cancer Stem Cells as Cells with Destabilized Gene Expression

As discussed above, gene expression variability (noise) is a source of cellular heterogeneity and phenotypic plasticity that are both hallmarks of embryonic and adult stem cells [101]. Variation in gene expression and the associated phenotypic heterogeneity account for randomness in cell fate decisions in stem and progenitor cells [44]. Moreover the degree of gene expression variability is modulated during development and differentiation: following a phase of highly widespread and variable gene expression, cells progressively transit towards more homogeneous, coordinated, and restricted gene expression profiles [38, 102, 103] associated with a more restrictive chromatin [104]. When hematopoietic stem or progenitors cells are induced to differentiate* in vitro*, a transient state characterized by an increased in gene expression variability is observed [102, 103] before its strong decrease.

Cellular interactions seem to be the major determinants in constraining and decreasing this expression variability and possess all the requirements to be considered as the main “constraints” leading to stable differentiated states. Various works on* Drosophila* or* Caenorhabditis elegans* embryos suggest that cellular communications and strong signaling are essential in stabilizing and homogenizing gene expression during cell differentiation [105, 106]. In mouse blastocysts, an initial phase of stochastic expression of individual genes precedes signal reinforcement through Fgf4 that segregates early lineages [107]. Also, direct cell contacts through gap junctions spatially coordinate prolactin gene expression in pituitary adult tissue [108], and disruption of gap junctions by enzymatic digestion or pharmacological inhibition reduced transcriptional coordination between cells [108], showing that perturbation of cell communication can enhance gene expression variability and phenotypic heterogeneity among differentiated cells. Thus, an alternative model of cancer where disruption of cellular interactions is the initial event by producing phenotypic plasticity and less differentiated cancer cells can be proposed [3, 92–94]. Basically, this tissue disruption would produce cells exhibiting increased epigenetic plasticity and increased gene expression variability. Thus CSC should be primarily defined as cells with such destabilized gene expression because they are no more in the normal cell-cell interaction network that stabilizes phenotypes and differentiation features in healthy tissues [3, 92–94].

This hypothesis has therapeutical implications. Indeed, if cancer cells remain intrinsically unstable and plastic because they do not normally interact with their native microenvironment, targeting driver genetic events is clearly not sufficient because this phenotypic instability and plasticity would allow them to counteract these treatments [93, 109]. On the contrary, molecules that stabilize gene expression and thus help to restore full differentiation and quiescence would be more prone to control cancer cell proliferation. As previously proposed as a general framework [109] and then specifically for multiple myeloma [94], only molecules able to interact with cancer cells and mimic their original microenvironment would be able to stabilize them. It should consist in pharmacological intervention with peptides or small molecules that mimic interacting domains of proteins that are part of the cell-cell interaction network in the healthy tissue. Nevertheless, as also previously suggested, this would not be sufficient. The reexpression through epigenetic drugs, for instance, of genes coding for proteins allowing these interactions would be an obligatory first step before introducing agents mimicking the native microenvironment. Indeed the subsequent stabilization of this reexpression would be achieved by bringing in their environment molecules that can interact with them [3, 109]. While there is no experimental evidence of the relevance of such a two-step pharmacological intervention, it could be speculated that it would improve the efficiency of epigenetic drugs that currently miss the second crucial step. A real differentiation therapy should consist in stabilizing the expression of the reexpressed proteins involved in differentiation and cell-cell interactions [109].

The role of gene expression variability in the appearance of therapeutic resistance independently of genetic variations is now demonstrated [110, 111]. Thus targeting this fundamental property of cancer cells, and especially of CSC, seems to be of major importance. Their plasticity in terms of gene expression and their ability to easily produce gene expression outliers with extreme expression levels and/or a large range of gene expression profiles have to be the privileged targets in anticancer therapy. And only concepts coming from recent developments in stem cell biology would help to define such therapeutic strategies.
